# Common Issues Seen in Paediatric Diabetes Clinics, Psychological Formulations, and Related Approaches to Management

**DOI:** 10.1155/2018/1684175

**Published:** 2018-02-27

**Authors:** Asma Deeb, Mariette Akle, Abdulla Al Ozairi, Fergus Cameron

**Affiliations:** ^1^Paediatric Endocrinology Department, Mafraq Hospital, P.O. Box 2951, Abu Dhabi, UAE; ^2^Medical & Surgical Psychiatry, Kuwait University, Kuwait City, Kuwait; ^3^Department of Endocrinology and Diabetes, Royal Children's Hospital, Melbourne, VIC, Australia

## Abstract

Diabetes is a chronic disease and its management is associated with multiple challenges. This is particularly the case in children and adolescents. Factors that contribute to difficulties in managing diabetes in youth include psychological characteristics, family dynamics, and social behavior. The purpose of this article is to highlight some psychological issues in children and adolescents with diabetes. We aim to present selected case scenarios encountered by health professionals and to provide tips on strategies for managing psychological aspect of diabetes. We tackle the psychological issues related to diabetes under four main categories: maladaptive disorders, eating disorders, family psychopathology, and family dysfunction. Psychotherapy and psychoanalysis are useful modalities in diabetes management. The psychological intervention is aimed at supporting patients and families to reach a balance between a normal family routine and a good glycemic control. We demonstrate unique requirements in coordinating care for children and adolescents with diabetes and highlight the importance of encouraging a positive behavior. Managing diabetes in children and adolescents needs to be in the form of a collaborative work between health care professionals, children and adolescents, and their families. Caring, supportive family backed up by experienced multidisciplinary team is the best approach to prevent psychological difficulties.

## 1. Introduction

### 1.1. Diabetes as a Unique Disease

Diabetes is a chronic disease and its management is associated with multiple challenges. The difficulty is more pronounced in the paediatric age group who has various requirements related to their growth and development. Factors that contribute to difficulties in managing diabetes in children include their status of growth and development, psychological characteristics and social behavior, health status in terms of diabetes control, family dynamics (including life style, socioeconomic status, and cultural beliefs), and care at nursery and school settings (Table 1).

### 1.2. Reaction to Diagnosis and Adjustment

The diagnosis of diabetes in a child can represent a crisis for children and family. Parents experience the classic stages of grief as they begin to understand the disease nature, the required management components, and the potential disease consequences [1].

Following diagnosis of diabetes, various negative reactions might be experienced by the child and parents. Symptoms of anxiety and depression might be normative adjustment responses as they tend to subside during the first year (case scenario III b) [2]. However, if the adjustment difficulties persist, there will be a greater risk for psychological issues [2]. Studies showed that family history of diabetes complications delays and adversely affects adjustment [3].

### 1.3. Impact of Diabetes on Mental Health

In younger school-age children, there have been reports of an increase of depressive symptoms after the first year of diagnosis. Over time, anxiety symptoms seem to decrease in boys but increase in girls [2]. In this study, school-age children were assessed over the first 6 years of their diagnosis and found a mild increase in depressive symptoms after the first year. Diabetes control is affected by the psychological status and presence of anxiety or depression symptoms. Higher A1C levels in adolescents have been associated with depressive symptoms, suggesting the importance of early diagnosis and treatment [4]. Despite the fact that many children and adolescents cope well with the demands of diabetes, there is a proportion that might end up having serious psychological problems including eating disorders and depression and experiencing recurrent ketoacidosis [5].

Harris and Lustman report that approximately one-third of patients with diabetes have diagnosable psychological problems at some point during their lifetime [6]. Psychological problems can range from affective problems, anxiety, and depression to eating disorders and behavioral changes, especially in children and adolescents. The authors also cite familial conflictual relations and authority issues among children and especially adolescents with diabetes. The latter tend to have a defiant attitude towards authority figures, including the health care professionals with their lists of daily requirements. Poor psychological support and defiant attitudes can lead to poor metabolic control and to diabetic ketoacidosis in some cases [6]. A study assessed 58 adolescents diagnosed with type 1 diabetes, and their parents with structured interviews concluded that youths with well-controlled diabetes who are encouraged to behave independently reported more cohesion and less conflict within the family [7].

### 1.4. Coping with Diabetes

Many factors influence the way the person copes with disease. These factors can be internal such as personality, coping mechanisms, and psychosomatic fragility or external in the form of family dynamics, social support, environmental circumstances, and quality of life. Both sets of factors influence the course of the disease management and the level of adaptation [8].

In a longitudinal study on mothers with children with type 1 diabetes, it was found that mothers cope better the longer their children had the illness [9]. Integration of diabetes care in the daily life of the person is to transform diabetes from being an “exterior object” in the person's life to becoming an integrated part of daily functioning. Care should be taken that the process of the integration be balanced allowing the “normal” life experience with minimal disruption of the smooth running of life routine.

### 1.5. DKA as a Sign of Family Dysfunction or Maladaptive Coping Strategy

Recurrent DKA can be a sign of family dysfunction. Studies showed that patients who lived in an environment with substantial family dysfunction might be at risk of repeated episodes of DKA [10]. Family dysfunction can be of various forms including inadequate parenting, single parenthood, financial stress, or lack of family involvement in diabetes management as demonstrated in case scenario I a.

Recurrent DKA can also be a result of insulin omission and represents a serious problem in the adolescent or the family. Insulin omission may be a maladaptive coping strategy. This behavior can be used as a manipulative way to get out of school or an abusive situation (case scenario I a) [11].

### 1.6. Approach to Adolescence

It is recommended that health care professionals acquire specialized counselling skills when dealing with adolescents. Essential tasks include persuading the adolescents to address their concerns, promoting a collaborative relationship and encouraging shared decision making. It is of high importance that mutual respect is emphasized. Addressing the adolescent's “number one” concern first is a good starting point.

Adolescents have difficulty adapting to diabetes as family communication and conflicts tend to deteriorate, and diabetes management often becomes the focus of parent-adolescent conflict [12]. The treatment burden pervades daily life, complicating other challenges of adolescence, and the regimen often becomes the focus of parent-adolescent conflict.

Parents who report letting go of perfectionism and keeping a positive attitude are more successful in attaining positive coping strategies [13]. Experts advise to encourage some level of ongoing parental involvement throughout adolescence, so applying interdependence rather than full independence [14]. This theory stems from the fact that pushing youngsters too hard to undertake diabetes management jobs can lead to adverse consequences. Adolescents who have diabetes management responsibilities too early at diagnosis might face an increased risk of treatment adherence and poor metabolic control [12].

### 1.7. Adherence Issues

During adolescence, adherence issues peak and metabolic control worsens [15]. This can be explained by the physiological changes and the increased insulin resistance. In addition, experimentation, rebellion, and risk-taking behavior are characteristics of adolescents which complicate to the adherence issue. Parents and professionals may use scare tactics by highlighting the threat of complications to motivate adolescents. This approach is mostly due to fear and frustration but it is unlikely to work. On the contrary, it can be counterproductive.

### 1.8. Eating Disorders

On applying the Diagnostic and Statistical Manual of Mental Disorders, 10%–15% of girls with diabetes meet the criteria for subclinical eating disorders including binge eating and insulin underdosing for weight control [16].

There are aspects of diabetes that may trigger the development of eating disorders. Insulin omission can be used to induce glycosuria leading to weight loss (case scenario II a) [11]. Initial weight gain on insulin treatment can be linked to insulin effect on weight gain by female adolescents, hence the tendency afterwards to omit insulin. Dietary restrain and restriction of favorable sweets can encourage extra insulin overdose to get the restrained sweet as a way of hypoglycemia correction (case scenario I b).

### 1.9. Family Dynamics

Family function in relation to health management is very important. Attitudes characterized by nagging and arguing are common reported strategies that increase repellence in adolescents. Ideally, family strategies should include goal setting, behavioral agreement, and social problem solving. All the latter strategies are reported to improve adherence in children with diabetes [5]. Family conflict, poor communication, and parental stress are indications for referral to mental health professionals (case scenario IV b).

Culture defines beliefs in various components in life including health, illness, responsibilities in maintaining health, family members' role in taking care of the person with illness, and decisions on treatment options [17]. People with diabetes need caring, warmth, support, understanding, and healthy limit setting. These criteria are best provided by a nurturing family environment [18]. Good metabolic control and a healthy psychological environment are strongly related [19]. Psychological well-being is influenced by metabolic control [20]. Socioeconomic status affects various aspects in diabetes. Low socioeconomic status has been reported to be related to increased prevalence of DKA, longer hospital admission, and worse diabetes control [21]. In addition to socioeconomic status, changes within families, including divorce and an increase in the number of work hours by parents, have decreased stability in the family. Having strong connection with a multidisciplinary team with skilled professionals promotes adherence to clinic visits and improves control in families with adverse social circumstances [22].

A child/adolescent personality and personal characteristics impact the success of diabetes management. Children with high self-esteem and competence tend to show better management of diabetes [14]. Social activities can be affected by the ability to handle peer pressure. Children with diabetes should be able to maintain normal school attendance and activities. Their school performance can be a good indicator of well-being and diabetes adjustment.

### 1.10. Psychological Support to Families with a Child with Diabetes

Psychotherapy and psychoanalysis can be useful modalities in diabetes management. As patients and families react to disease adaptation differently, each patient/family should be taken individually. Although there are general therapeutic rules to manage a specific disease, customizing approach is always necessary to fulfill individual needs and characteristics.

Randomized controlled trials in children and adolescents with type 1 diabetes showed that psychological therapy including counselling, cognitive behavior therapy, family systems therapy, and psychodynamic therapy improve glycemic control in children and adolescents [23].

In summary, diabetes is a disease that is commonly associated with maladaptive disorders [24]. Eating disorder is another common issue in adolescents with diabetes and is associated with poorer glycemic control [25]. Other psychopathology forms are also seen in diabetes including pathological anxiety or fear and depression, and not all patients are prepared to engage and accept therapeutic intervention [26]. Family functioning is directly related to adherence behavior. It is reported that family dysfunction is seen more commonly in children who are nonadherent to diabetes therapy and have poor glycemic control [27].

### 1.11. Aim

The purpose of the article is to highlight some psychological issues in children and adolescents with diabetes. We also aim to present selected case scenarios encountered by health professionals and to provide tips on strategies for managing psychological aspects of diabetes.

The case scenarios presented below are divided under the four categories: maladaptive disorders (I a–c), eating disorders (II a), family psychopathology (III a, b), and family dysfunction (IV a, b).

## 2. Maladaptive Disorders

### 2.1. Case Scenario Discussion

#### 2.1.1. Scenario I a: A Male Adolescent Who Is Noncompliant with Glucose Monitoring and Insulin Injection

Ali is a 16-year-old boy who was diagnosed with type 1 diabetes since the age of 8 years. He is a bright boy who is capable of injection and monitoring and has a good knowledge of diabetes and its management. Ali's diabetes control is poor with an HbA1c between 9–12% since his early diagnosis. He had multiple admission for DKA. He admits to not being regular on injections and he tests once daily at most. There has always been a lack of family involvement in Ali's diabetes management. Being a bright young man, his parents have always assumed that he is able to take care of his diabetes on his own.

### 2.2. How Would You Approach Him?

#### 2.2.1. Psychological Formulation

Ali is an adolescent who had diabetes for 8 years. He is going through adolescence crisis and is wishing for a normal diabetes-free life. While he is building his identity and is projecting himself in his future plans, he gets angry and frustrated with diabetes that he needs to live with. His noncompliance with diabetes management is not due to lack of education or knowledge. Rather, it stems from the emotional resistance to accepting living with a chronic disease.

#### 2.2.2. Approach

The first step in the approach is to listen to his experience with diabetes, his effort, his feelings, and his representations of diabetes. It is important to validate and acknowledge feelings, frustration, and fatigue. In the meantime, acknowledging the reality is equally important in the sense that diabetes is a chronic disease, and it needs to be managed properly to avoid complications. Dealing with the reality can be difficult for adolescents. However, even implementing small and gradual changes can be significant. Setting specific, feasible, and attainable goals enables reaching the target. For such a scenario, a “negotiation approach” might be useful. If the target is to check blood glucose 4 times per day, then agreement to start by doing 2 per day might be acceptable. Ensuring that the diabetes team will see and discuss those blood glucose checks in the next visit is important as it enforces the improvement in compliance. The team should, at this stage, concentrate on highlighting the improvement of the monitoring frequency. Gradually, treatment is adjusted based on the profile and improvement is constantly highlighted and acknowledged. This way, he will realize that the change he initiated in increasing monitoring frequency is rewarded by the acknowledgment of the team and ultimately by the improvement in glucose profile. Of vital importance in such a case scenario is involving the family. While self-dependence in diabetes management is encouraged; family support and supervision are proven to have a significant influence on metabolic control [28].

#### 2.2.3. Scenario I b: A School Child Who Inflicts Hypoglycemia by Repeated Insulin Injections to Be Offered Sweets/Chocolate

Ahmed is a 10-year-old bright boy who was diagnosed with diabetes 9 months ago. He has a good understanding of diabetes. His parents comment that he loves sweets and sugary drinks and is fond of chocolate. He has gone through a period of honeymoon which lasted around 4 months. His parents note that he has repeated hypoglycemia with marked reduction of insulin requirements. None of the hypoglycemia episodes has been reported to be due to injecting insulin then not eating the planned meal. Ahmed needed treatment with sweets and juices for hypoglycemia on regular occasions. Doctors suspected self-inflicted hypoglycemia by taking extra insulin dosages and discussed with Ahmed who admitted this practice.

### 2.3. How Would You Approach Him?

#### 2.3.1. Psychological Formulation

Ahmed's manipulation of diabetes care can be a way to overcome diabetes food restrictions. Inducing hypoglycemia might be an indication of the difficulty he has accepting diabetes care restrictions. It could also be a way of dealing with frustration about food restrictions. He is not refusing care but he is not accepting the restrictions of sweets which he enjoys having.

#### 2.3.2. Approach

Ahmed's manipulation could be an expression of his parents' anxiety and inflexible control. Attention should be paid to the level of restrictions imposed by the parents. If the parental control imposes complete banning of sweet, it might create a state of repulsion by Ahmed. The repulsion can then be expressed through manipulation and inflicting hypoglycemia which predispose him to self-harm.

It is advisable that discussion with Ahmed and his parents be around “allowances” of reasonable amount of sweets that he can have rather than a complete ban. Agreement can be drawn on the frequency and the amount of sweet he can have. Suggestion of timing after main meals can provide a good option as insulin to cover the extra carbs in the sweet can be added instead of an extra separate injection to cover the sweet. It is important to explain to Ahmed that sweets are unhealthy option of food, and the restriction should be applied to everybody including those who do not have diabetes particularly siblings and friends.

It is important to counsel parents on the appropriate dietary control and tackle anxiety about sugary food intake. Involving education and reassurance on the positive behaviors and care requirements are useful components in the counselling process. In addition, advising on the importance of having a more comprehensive, less-anxious, and less-controlling approach will help the family achieve a healthy balance between proper diabetes management and avoidance of complete dietary restrictions. Creating this balance might help Ahmed to have the freedom of enjoying what his siblings and peers enjoy, while achieving proper diabetes control and treatment adherence. Equally important, the family should be advised to monitor and supervise Ahmed's insulin injections and consumption. Unexpected early need for renewal prescriptions and refilling insulin supply should alert them to the excessive use of insulin.

#### 2.3.3. Scenario I c: A Child Who Fakes Glucose Readings in the Log Book

Adam is a 12-year-old boy who is diagnosed with type 1 diabetes for the last 3 years. He participates effectively in his diabetes management. He is a “perfectionist” boy who is doing very well at school and is the eldest of his 3 other siblings. Adam records his glucose reading in a log book and takes it with him to the clinic but does not bring the glucometer. His log book always looked neat and tidy with complete record of perfect readings. His HbA1c in the latest visit was 10.3%. Doctors requested his mother to bring the glucometer which showed marked discrepancy of readings compared to Adam's log book readings. The issue of the result discrepancy was highlighted to Adam and his parents and he admitted writing unreal numbers in his log book.

### 2.4. How Would You Approach Him?

#### 2.4.1. Psychological Formulation

Being a perfectionist, one can assume that he cannot cope with having poor diabetes control. As he might fear judgement and negative results, he deals with the situation by falsifying readings, a behavior that can be classified as autodestructive. Adam comes to the hospital with fear of “being judged” in mind. He might be thinking that diabetes management is another test he should pass well, as he does at school. As diabetes management is challenging, he feels that he might fail the test which he does not accept. Falsified readings reassure him in his abilities to pass the test. Adam is experiencing diabetes care as another coercive obligation with pressure from the parents and the team that he needs to tackle. He desires to have a “normal diabetes-free” life and is trying to save himself from failing perfection (which he is used to) by providing an ideal but fake profile. Another possible reason for falsifying numbers may simply be to lead parents or physicians to assume he is testing, when in fact, he is not. This behavior can reflect showing perfection in management and testing frequency.

#### 2.4.2. Approach

Counselling Adam can begin by acknowledging his feelings and emotions including fatigue, frustration, and anger. Acknowledgment of the difficulty in diabetes management should also be done. He needs to be congratulated on his efforts, but he needs to understand that he does not necessarily need to be perfect “all the time.” He needs to understand that falsifying readings is harming him as it leads to incorrect insulin dosing and improper diabetes management.

It is advisable that the team avoid blame for not reaching the target. However, in a target-orientated approach, certain amount of judgement of passing or failing the target should be acknowledged. A plan for reaching the target should, then, be agreed to achieve better care and improve glycemic control.

The parents should be counseled on the importance of understanding Adam's feelings and not putting much pressure on him. Counselling Adam and his family that fluctuation in glucose profile can be expected in type 1 diabetes might help them understand the natural disease process and its proper management. Helping Adam incorporate diabetes care routine into his daily routine and taking small steps towards diabetes management might help him achieve better adherence, without inflicting extra pressure on him.

## 3. Eating Disorders

### 3.1. Scenario II a: A Female Adolescent Who Is Skipping Insulin Fearing Weight Gain

Sarah is a 15-year-old girl who has diabetes for the last 4 years. She is on multiple injection of insulin and is the one who injects her own insulin. She is very conscious about body image and anxious about weight gain. Sarah skips injections fearing that insulin will increase her weight. She has also been skipping meals and has been on a minimum carbohydrate containing-diet. Her diabetes control is poor with HbA1c above 9%. Sarah's mother had a history of binge eating and was on a long dieting program. She tends to center diabetes control for Sarah on food and weight which has created tension and lack of trust between Sarah and her mother.

### 3.2. How Would You Approach Her?

#### 3.2.1. Psychological Formulation

Sarah's concern about weight gain is likely to be related to adolescent period and changes. She is mixing her weight control with her diabetes management. Manipulating her diabetes management for other purposes indicates possible underlying issues related to self-image. She might have a distorted self-image which is pushing her to acquire an eating disorder through a self-destructive behavior. In addition, her mother's history of binge eating might have an impact on her attitude to food and dieting in general. It is shown that maternal weight issues and mother-daughter relationship contribute to eating disturbances in girls with diabetes and that might be the case here [29].

#### 3.2.2. Approach

Sarah's concern should be addressed during psychotherapy sessions. Exploring body image disturbance is required and if it exists, it might be helped by psychotherapy. It is recommended that Sarah is counselled that skipping insulin and manipulating her daily care are not the best way for weight loss and it might cause harm. Educating Sarah on the routine care, the important role of insulin, the positive behaviors, and the goals of care is of paramount importance. Effects of manipulating treatment should be highlighted as well as possible consequences on health. Follow-up and regular support are crucial to help her find other solutions for her weight concern and treat underlying problem. In addition, counseling can offer Sarah alternatives to weight loss that also help control her diabetes through measures of controlling diet and regular physical activity.

## 4. Parental Psychopathology

### 4.1. Scenario III a: A Mother Who Gives Extra Night Snacks to Prevent Nocturnal Hypoglycemia

Fatima is a 3-year-old girl who was recently diagnosed with diabetes and started on insulin injections. Although her parents coped with the diagnosis and managed Fatima very well, her mother remained scared of nocturnal hypoglycemia. Her routine was giving Fatima extra snacks before bed to ensure blood glucose above 200 mg. This resulted in persistent hyperglycemia overnight and a high HbA1c. Her mother says, “it is better to have highs than lows.”

### 4.2. How Would You Approach This Scenario?

#### 4.2.1. Psychological Formulation

Fatima's mother's behavior is probably resulting from anxiety over hypoglycemic episodes. Her “inducing hyperglycemia” routine is an attempt to prevent hypoglycemia at nighttime. She is new to diabetes management and her fear can be part of the adjustment process.

#### 4.2.2. Approach

Counselling her on the adverse effects of hyperglycemia resulting from such routine is necessary. It is important that the mother understands that giving routine snacks before bed will induce hyperglycemia and elevates the HbA1c. Educating the mother on the proper way of monitoring and preventing nocturnal hypoglycemia is most helpful, and reassuring her on the safety of the positive behavior is important. Achieving her acceptance of the concept of the proper management of preventing hypoglycemia will eventually lead to improving behavior and adherence to the proper management.

If resources are available, discussing continuous glucose monitoring devices equipped with hypoglycemia alarms might be beneficial in alleviating the anxiety and fear.

### 4.3. Scenario III b: A Father with Diabetes Who Has Guilt Feeling of Transmitting Diabetes to His Daughter

Lara is a 13-year-old girl with poorly controlled diabetes. Her father also has type 1 diabetes. Since Lara's diagnosis, her father became withdrawn and progressed to clinical depression. He had constant guilt feeling that he has transmitted the disease to her, and his diabetes is the reason that Lara developed the same disease. Lara has always felt sad about her father's withdrawal, and she too developed guilt feeling and symptoms of depression.

### 4.4. How Would You Approach This Scenario?

#### 4.4.1. Psychological Formulation

Guilt feeling is frequently expressed by adolescents with diabetes and psychological therapy can be effective in its management [30]. The guilt feeling in adolescents with diabetes might arise from their beliefs that they can be a “burden” on their family. In Lara's case, the impact of her disease on her father's psychological status resulted in acquiring a set of feelings and representations which might have adversely affected her diabetes control.

#### 4.4.2. Approach

The team's educational intervention should aim at explaining the etiology of type 1 diabetes to Lara and her father and clarifying that it is not inherited on genetic basis as other autosomal diseases. Accordingly, he should not feel that he is responsible for the “disease transmission.” It is suggested that Lara and her father be offered psychotherapy to help them deal with the emotional burden of guilt and sadness. Medical and scientific facts are helpful to address any misconceptions and beliefs which might help Lara and her father to better understand the disease and alleviate the guilt feeling.

## 5. Family Dysfunction

### 5.1. Scenario IV a: Diabetes in a Girl Resulting in Adverse Impact on Family Dynamics and Parents-Children Relationships

Tamara is a 10-year-old girl who was diagnosed with diabetes three years ago. She has an excellent glycemic control with a HbA1c ranging between 7.0 and 7.5%. Her parents are dedicated to her daily diabetes management and are very compliant with tasks, such as injecting insulin, testing blood glucose, supervising diet intake, and correcting hyperglycemia. Her twelve-year-old sister has repeatedly complained of the “extra care” their parents are giving to Tamara and started arguing that they do not spend as much time with her.

### 5.2. How Would You Approach Such a Scenario?

#### 5.2.1. Psychological Formulation

Tamara's case is typical of the situations where diabetes impacts the family dynamics and parents-children relationships. Tamara's sister is probably jealous, and this jealousy is accentuated by the extra care imposed by diabetes requirements for Tamara. Tamara on the other hand is having the extra care and attention because of her diabetes. While her other needs, desires, and other life aspects are being overlooked by diabetes care requirements, so are her sister's needs. Such parental attitude can stem from a profound anxiety about diabetes and is intended to control diabetes.

#### 5.2.2. Approach

Psychotherapy can help parents to discuss their anxiety and fear. It helps to address the excessive care routine that might become exhausting and impact the quality of life of the parents reflecting on the care for their children. It can also address the importance of remaining “the parents of Tamara and her sister” and not the “parents of a diabetic child.”

Taking into consideration the diabetes requirements while keeping in mind the child's regular needs will help them find some balance between the 2 aspects of their care. Acknowledging Tamara's sister's needs and explaining to her the diabetic needs of Tamara are steps towards a better acceptance and possible involvement in her care. The family might be able to develop a care system that involves all members and takes into consideration their different needs. A balance can be achieved to reorganize the functions in the family, without placing diabetes as its utmost center.

### 5.3. Scenario IV b: A Child with Diabetes Living in a Dysfunctional Family Settings

Yassin is an 8-year-old boy who has a poorly controlled diabetes. He misses insulin injection regularly, does not monitor his glucose profile, and eats sweets and sugary drinks liberally. His parents are continuously fighting over his diabetes management. His father constantly accuses his mother of being negligent of the diabetes management. Yassin lives in an extended family. The family practices food/sweet reward routines which Yassin refuses to be exempted from. His mother is finding it difficult to persuade his grandparents of avoiding giving him sweet which they find it a way of showing their love and affection. His cousins tease him when he is not joining in eating the sweets, so he eats with them to prove that he can be “normal,” too.

### 5.4. How Would You Approach This Scenario?

#### 5.4.1. Psychological Formulation

The whole family setting, the conflict between the parents, the extended family culture, the food reward rule, and the lack of proper care and limits are propitious for a child to live by his desires and satisfy his eating whims without any restrictions. The main issue is in the parental role, guidance, and supervision that are lacking.

The fight over his diabetes and the constant accusation by the father to the mother could be an indicator of underlying marital issues and possibly conflict that is being “externalized” on the son's diabetes. Parents could be using diabetes as a reason for conflicts with the child being objectified. This attitude is typical of familial dysfunction. Living in the large family multiplies the authority/influencing figures extending it to grandparents, uncles, aunts, cousins, and other children.

#### 5.4.2. Approach

The adult family members have a responsibility of supporting the parents in their care of the child. The team can support the parents with improving knowledge and education. Positive behaviors and goals of care need to be explained to the parents aiming to change the family attitude and dynamic towards diabetes management.

The psychotherapy is intended at addressing the couple's issues and helping them solve their conflicts separately from their child diabetes care. Parents need to understand that the liberty to enjoy unhealthy eating habits is at the cost of his health. In addition, he will grow up neglecting his diabetes, will not be able to discern positive behaviors from unhealthy habits, and will not have the proper self-care skills that are needed for a lifetime in the case of diabetes.

## 6. Conclusion

Diabetes is a chronic disease that imposes a heavy burden on families with children with diabetes. The cases presented demonstrate unique requirements in coordinating care for children and adolescents with diabetes. The characteristic features of children related to their growth and development illustrate how diabetes is a complex disease in the young. Managing diabetes in children and adolescents is a collaborative work between health care professionals, children and adolescents, and their families. Caring, supportive family backed up by experienced multidisciplinary team can be the best approach to prevent psychological difficulties. Education and treatment option need to be prioritized and positive behavior changes encouraged.

## Figures and Tables

**Table 1:** Various developmental aspects and their impact on diabetes management.

Developmental change	Results	Possible impact
Physical	(i) Small body size (ii) Rapid growth (iii) Progress in developmental milestone	(i) Small insulin doses, might need insulin dilution (ii) Close follow-up for adjustment of doses

Behavior	(i) Erratic eating behavior (ii) Altered sleep pattern	(i) Need of multiple insulin dosing (ii) Risk of insulin stacking (iii) Inconsistency of meal timing (iv) Flexibility on glucose monitoring is required

Moral	(i) Judgment based on personal preference	(i) Difficulty in cooperation with treatment form of injection and monitoring

Emotional	(i) Sense of self and others	(i) Acceptance of carers providing the treatment might be difficult

Cognition & comprehension	(i) Understand dynamics and surrounding (ii) Acquire language (iii) Learn and expand expression	(i) Expression of symptoms (ii) Expression of wish or refusal of treatment

Social	(i) Interactive play (ii) Playgroup and preschool attendance	(i) Involvement of other personnel in the care of diabetes

Role & responsibility	(i) Majority of care by parents with variable involvement	(i) Gradual involvement of care

Incentive concept	(i) Immediate incentive expectation	(i) Possible expectation of regular rewards to routine management procedure

Learning	(i) Acquiring physical and mental skills	(i) Need to adapt a management change in parallel to the developed skills
